# Common Salt in Scarlet Fever

**Published:** 1852-05

**Authors:** 


*Common Salt in Scarlet Fever.*

In a former number of this periodical, a number of cases were reported illustrative of the beneficial effects of common salt in intermittent and remittent fevers. The following, from the Boston *Med. and Surg. Journal*, shows it is also a valuable remedy in another class of diseases:

Dr. H. A. Ramsay, of Georgia, writes to us that he has used in Southern scarlet fever, in every grade, an emetic of the common table salt. "It is far superior," he says, "to all other remedies of the emetic class; indeed, in my conception, it seems to exert a specific effect upon the disease. The medicine is quite harmless in its operation, and may be repeated with impunity, *pro re nata*. Have you New England physicians ever tried the remedy? If so, with what success? I imagine the scarlet fever of New England is much more *malignant* than in our country."

